# Draft Genome Sequence of *Streptomyces* sp. TP-A0356, a Producer of Yatakemycin

**DOI:** 10.1128/genomeA.01446-15

**Published:** 2015-12-10

**Authors:** Hisayuki Komaki, Natsuko Ichikawa, Akira Hosoyama, Nobuyuki Fujita, Yasuhiro Igarashi

**Affiliations:** a Biological Resource Center, National Institute of Technology and Evaluation (NBRC), Chiba, Japan; b NBRC, Tokyo, Japan; c Biotechnology Research Center and Department of Biotechnology, Toyama Prefectural University, Toyama, Japan

## Abstract

Here, we report the draft genome sequence of *Streptomyces* sp. TP-A0356, a producer of a potent antitumor antibiotic, yatakemycin, to evaluate potential for secondary metabolite production. The genome sequence data suggest the presence of at least nine gene clusters for polyketide synthases and nonribosomal peptide synthetases in this strain.

## GENOME ANNOUNCEMENT

Yatakemycin is the most potent member of a class of DNA-alkylating agents that also includes CC-1065 and duocarmycins (1, 2). The complete sequence of the yatakemycin biosynthetic gene cluster and the biosynthetic pathway has been reported (3). As a part of our screening for novel secondary metabolites through a genome mining approach (4), the yatakemycin-producer *Streptomyces* sp. TP-A0356 was subjected to the whole-genome shotgun sequencing to survey polyketide synthase (PKS) and nonribosomal peptide synthetase (NRPS) genes.

*Streptomyces* sp. TP-A0356 was deposited to the NBRC culture collection (NBRC 110464). The genomic DNA of the strain TP-A0356 monoisolate was sequenced by a combined method of shotgun sequencing using 454 GS FLX Titanium (Roche) and paired-end sequencing with MiSeq (Illumina). A hybrid assembly of the 454 GS FLX Titanium data (73 Mb, 8.7-fold coverage) and the Illumina paired-end data (774 Mb, 92-fold coverage) was performed with Newbler version 2.8 (Roche). Contigs obtained from the assembly were subsequently finished using GenoFinisher (5). The resulting scaffolds were analyzed using Prodigal (6) for the prediction of protein-coding genes. The draft genome sequence of strain TP-A0356 consists of 62 scaffolds with a total size of 8,409,357 bp, 101-fold sequencing coverage, and 70.8% G+C content.

PKS and NRPS gene clusters were searched for in the same manner as previously described (7). The genome contains at least five type I PKS gene clusters, one type II PKS gene cluster, and three NRPS gene clusters. Four type I PKS gene clusters were divided into scaffold-12 and -21, scaffold-14 and -47, scaffold-37, -48, and -55, and scaffold-56, -58, and -59. The PKSs in scaffold-12 and -21 show 74 to 94% sequence identities to polyunsaturated fatty acid synthases (PfaA and PfaC) of *Streptomyces* sp. SPB74. Two PKSs, orf1 and orf2 in scaffold-37, show about 60% sequence identity to SalB and SalA, respectively, for salinomycin synthesis (8). The type II PKS gene cluster in scaffold-3 is speculated to be involved in spore pigment production. The NRPS gene clusters have only a single module, indicating that their products are small peptides. Most of the PKS and NRPS gene clusters do not show high similarities to gene clusters of known compounds. These bioinformatic analytical data suggest the potential of strain TP-A0356 to produce unknown metabolites of type I PKS origin.

### Nucleotide sequence accession numbers.

The draft genome sequence of *Streptomyces* sp. TP-A0356 has been deposited in the DDBJ/ENA/GenBank database under the accession no. BBZJ00000000. The version described in this paper is the first version, BBZJ01000000.
